# School Principals’ Stress Profiles During COVID-19, Demands, and Resources

**DOI:** 10.3389/fpsyg.2021.731929

**Published:** 2021-12-16

**Authors:** Katja Upadyaya, Hiroyuki Toyama, Katariina Salmela-Aro

**Affiliations:** Faculty of Educational Sciences, University of Helsinki, Helsinki, Finland

**Keywords:** stress, latent profile analysis (LPA), school principals, COVID-19, demands and resources

## Abstract

The present study examined latent profiles of school principals’ stress concerning students’, teachers’, parents’, and principals’ own ability to cope during the COVID-19 pandemic. In addition, the role of job demands (workload, remote work stress, difficulty to detach from work, COVID-19 crisis, COVID-19 infections at school, impact of COVID-19 on future teaching), resources (buoyancy, effective crisis leadership, social appreciation, successful transition to remote teaching), and occupational well-being (measured as job burnout and engagement) in predicting the latent profiles of stress sources was examined. The participants were 535 (59% women) school principals across Finland, who answered to a questionnaire concerning their sources of stress and occupational well-being during spring 2020. Three latent profiles were identified according to principals’ level of stress: *high stress* (41.4% of the school principals), *altered stress* (35.9%), and *low stress* (22.7%) profiles. Work burnout, workload, COVID-19 related concerns, and difficulty to detach from work increased the probability of principals belonging to the high or altered stress profile rather than to the low stress profile. Work engagement, buoyancy, and social appreciation increased the probability of principals belonging to the low rather than to the high or altered stress profile.

## Introduction

At the beginning of 2020, SARS-CoV-2 (COVID-19) virus spread across the world and disrupted teaching and learning of millions of students across the globe. At the onset of the pandemic school principals had to respond to school closures and fundamental shifts in education rapidly, one of the major changes being shifting from regular in-person learning to remote learning and teaching (Weiner et al., 2021). At schools and different educational institutions principals were leading the transitions to remote teaching and learning, and they were tasked with helping teachers and staff, students, and their parents to adjust to the new continuously changing environment (Weiner et al., 2021). Simultaneously, increasing concerns about the spread of the virus and concerns about family members, friends, colleagues, and students and their families getting sick were present, which might have altered school principals’ level of stress. Previous research has indicated that such concerns related to the school community are often among the most prevalent stressors in school principals’ work (Elomaa et al., 2020, 2021), and high stress may limit principal effectiveness in times of crisis (DeMatthews et al., 2021). However, even less studied, it is possible that individual differences exist in school principals experiences of stress concerning the school society’s ability to cope with the pandemic. For example, it is possible that during COVID-19 subgroups of principals experienced high/altered/low stress concerning the school community’s ability to cope with the COVID-19 crisis (see also Innanen et al., 2014; Salmela-Aro et al., 2019). These differences can be captured with person-oriented research, such as latent profile analysis (LPA). Moreover, due to COVID-19, principals had to face several new demands (e.g., remote work demands, impact of COVID-19 on future teaching) related to the unexpected situation, which may have altered their stress concerning the school community’s ability to cope. Consequently, using the job demands-resources framework (Demerouti et al., 2001; Bakker and Demerouti, 2006, 2017), the present study examines latent profiles of principals’ stress concerning the school community’s (e.g., students, teachers, parents, principals) ability to cope during COVID-19 pandemic, and the associations between the latent profiles and principals’ job demands (e.g., workload, remote work stress, difficulty to detach from work, COVID-19 crisis, COVID-19 infections at school, impact of COVID-19 on future teaching), resources (e.g., buoyancy, crisis leadership, social appreciation, schools’ adaptation to remote learning), job burnout and engagement.

### Principals’ Stress Concerning the School Community’s Ability to Cope With COVID-19 Pandemic

Even job satisfaction among school principals is often high, many principals simultaneously experience altered levels of occupational stress (Darmody and Smyth, 2011). One of the main sources of their stress is principals’ concerns about students, school climate (Darmody and Smyth, 2011), and interpersonal-, and health concerns (Elomaa et al., 2021, see also Dicke et al., 2018a). For example, principals who work in schools where over a quarter of students report altered emotional/behavioral problems experience higher occupational stress compared to schools where such problems are less prevalent (Darmody and Smyth, 2011). Spread of COVID-19, and psychological and financial strain that the pandemic caused among many families likely increased principals’ concerns about the school community and how students, parents, and teachers were coping with the unexpected situation. In Finland most schools were closed nearly 2 months during the initial phase of COVID-19 in spring 2020. Social interactions were heavily reduced and lockdowns, quarantines, and physical distancing measures took place, causing several turmoil in many people’s lives. Students were not able to meet with their peers, and both teachers and students were forced to switch to remote learning following a rapid schedule. School principals were leading these sudden changes in the school, responding to changing regulations the government proposed, and helping the school community to adjust to the continuously changing environment. All these changes combined with worries about the spread of the virus and the psychological impact of the pandemic likely altered principals’ concerns about the school community’s ability to cope. Even less studied, some individual differences may also take place in principals’ experiences of stress. For example, it is possible that among some principals concerns about students’ coping were highlighted, whereas other principals were more concerned about teachers’ and parents’ ability to cope. Thus, the present study was among the first to examine latent profiles of principals’ stress concerning the coping of the school community during COVID-19.

### Job Demands and Resources

The demands-resources (JD-R) model (Demerouti et al., 2001; Bakker and Demerouti, 2006, 2007, 2017), describes various demands and resources which are present at workplace, and often antecede employees’ experiences of job-related stress. Both demands and resources can be described in terms of personal, social, and job environmental aspects of work. Job demands require sustained physical/psychological effort and include related costs (Demerouti et al., 2001). In school principals’ job, workload is a typical demand, which is characterized by completing multiple tasks, and requires extended mental effort. During COVID-19, multiple unprecedented personal demands (remote work stress, strain caused by the COVID-19 crisis) and social demands related to the school community (COVID-19 infections at school, impact of COVID-19 on future teaching) emerged due to the rapid changes in the school environment. Due to the altered concerns about the school community’s coping with the pandemic and intensification of new work demands, some principals might have found it also difficult to detach from work while attempting to respond to the crisis situation. Psychological detachment from work describes individuals’ ability to disengage during off-work hours, which is an essential part of recovery (Sonnentag, 2012). Inability to detach and “switch off” from work during leisure time can later manifest as altered occupational stress (Sonnentag, 2012).

Job resources, in turn, help employees to face the challenges presented by job demands and reduce the related physiological/psychological costs, function as a tool to aspire work-related goals, and stimulate one’s personal growth and development (Demerouti et al., 2001). Job resources are often beneficial for one’s job-related well-being and help in reducing job-related stress (de Jonge et al., 2008; Upadyaya et al., 2016; Upadyaya and Salmela-Aro, 2020). One important personal resource is buoyancy, which refers to one’s beliefs on how much control they have over their work and the work environment (Bakker and Demerouti, 2017), and how able one is to successfully overcome challenges and setbacks that occur at work (Martin and Marsh, 2008b; Parker and Martin, 2009). Individuals who have high personal resources often have faith in their own capabilities to face unforeseen events (Bakker and Demerouti, 2017), such as sudden changes at work related to a pandemic. During the pandemic principals’ crisis leadership as a personal resource, as well as school community’s adaptation to remote learning and appreciation of principals’ work (social resources) became highlighted. At the onset of COVID-19, a rapid response from school principals was essential for effective crisis management, which included quick assessment of the situation, fast decision making under uncertain circumstances, creating new strategies, and making radical changes in the school community (Fernandez and Shaw, 2020). Fast adaptation to the remote teaching and learning facilitated the continuation of schooling, and social support from the school community may have buffered against the negative influence of job demands (Bakker et al., 2007), and protected principals against higher levels of stress.

**FIGURE 1:**
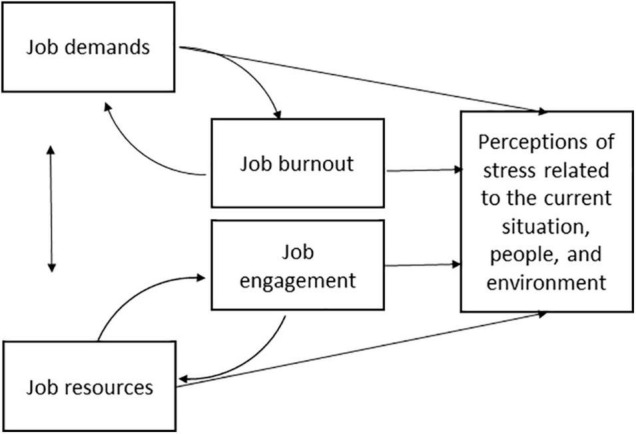
Model of the associations between job demands, resources, occupational well-being and current stressors.

In addition to demands and resources, occupational well-being may have affected the level of stress principals experienced during the pandemic. Occupational well-being is a broad construct covering multiple dimensions and definitions (Kowalski and Loretto, 2017). In the present study, occupational well-being was described in terms of job burnout (e.g., exhaustion, cynicism and feelings of inadequacy at work) and engagement (e.g., absorption, energy, and dedication at work), a definition often used studies within the job demands-resources framework (Schaufeli et al., 2002; Näätänen et al., 2003; González-Romá et al., 2006; Dicke et al., 2018b). According to the JD-R theory (Bakker and Demerouti, 2007, 2017), these well-being factors affect the level of job demands and job resources, which, thus, suggests that they predict the level of perceived stress raising from various sources. Consequently, it is possible that together with job demands and resources occupational well-being factors such as job burnout and engagement predict employees’ perceptions concerning stressors related to the current situation (as presented in Figure 1). The present study examined further the associations between demands, resources, well-being, and principals’ stress concerning the school community’s coping during the COVID-19 pandemic.

## Aims

The present study aimed at investigating the following research questions:

1) What kind of distinct latent profiles (e.g., groups of homogeneous subjects) can be identified according to principals’ stress about students’, teachers’, parents’, and principals’ own ability to cope during COVID-19? Due to the explorative nature of the analysis, we have to be cautious about precisely formulating hypotheses on the number of latent profiles. However, based on previous research (Innanen et al., 2014; Salmela-Aro et al., 2019), we expect to find at least two distinct profiles, one representing school principals who report high stress, and another profile representing a low stress profile.
2) To what extent principals’ demands (workload, remote work stress, difficulty to detach, COVID-19 crisis, COVID-19 infections at school, impact of COVID-19 on future teaching), resources (buoyancy, crisis leadership, social appreciation, adaptation to remote learning), and job well-being (burnout and engagement) are associated with principals belonging to different stress profiles? Based on some previous research findings (Upadyaya et al., 2016; Salmela-Aro et al., 2019), we expect that job demands are positively associated with principals belonging to higher stress profiles, and that resources are associated with principals belonging to lower stress profiles.

## Matrials and Methods

### Participants

This study is part of the school principal barometer, which follows up school principals annually across Finland. The present study uses data from the second wave which was collected in spring 2020 during the COVID-19 pandemic. An online survey was distributed to 1,200 school principals (response rate 54%) via email concerning their job-related stress, demands, resources, and well-being. Altogether 535 (59% women) principals participated the study. Most principals had a Master’s degree (95.6%). The age range of the participants was 29–40 (11%), 41–50 (35%), 51–60 (45%), and 61–66 (9%) years old. Participation in the study was voluntary and the research project was approved by the Ethical Review Board of the University of Helsinki and the research protocol followed their guidelines.

### Measures

*Stress sources* were measured with four questions (adapted from Dicke et al., 2018a) concerning school principals’ stress over the school community’s ability to cope with the COVID-19 crisis (“The following questions concern stressors at your leadership position. Please estimate how stressful the following factors have been for you during the past 3 months.” “Students’…; teachers’…; parents’… my own ability to cope.”). The scale anchors were 1 = minor source of stress; 10 = significant source of stress.

*Job demands* were measured in terms of *workload, difficulty to detach from school* (see also Dicke et al., 2018a), *COVID-19 crisis* [“On a scale 1–10, please estimate how stressful the following factors have been during the past 3 months: (a) workload, (b) COVID-19 crisis, (c) difficulty to detach from school community.”; 1 = minor source of stress; 10 = significant source of stress], *remote work stress, COVID-19 infections at school, and impact of COVID-19 on future learning* [“On a scale 0–10: (a) how much remote work has increased your stress?, (b) how concerned you are about the COVID-19 infections at your school community, (c) how much you think COVID-19 will affect teaching after the pandemic?; 0 = not at all; 10 = a lot; see also Dicke et al., 2018a].

*Job resources* were measured in terms of *buoyancy* (3 items (Martin and Marsh, 2008a), “I am good at dealing with work pressures.”; 1 = not at all true; 7 = very much true; Cronbach’s α = 0.85), principals’ *COVID-19 crisis leadership efficacy* (C-LEAD; 6 items, “Think about your actions during the COVID-19 crisis. What do you think about the following statements? – I can make decisions even under extreme deadlines.”; 1 = completely disagree; 5 = completely agree; Cronbach’s α = 0.85; Hadley et al., 2011), school community’s *adaptation to remote learning* (6 items; “How well did your school/teachers/students/parents/schools’ food supply/special education succeeded in the transition to remote learning?”; 1 = very poorly; 5 = very well; Conbach’s α = 0.85), and *social appreciation* (3 items; “Does your schools’ personnel/executive team/municipals’ educational administration value the work you do as a leader in the face of the COVID-19 crisis?”;1 = always; 4 = never (reversed); Cronbach’s α = 0.72). All sum scores were formed with the mean function.

*Job well-being* was measured in terms of job burnout and engagement. *Job burnout* was measured with the Bergen Burnout Inventory (Näätänen et al., 2003; Salmela-Aro et al., 2004, 2011) which consists of 15 items measuring exhaustion at work (e.g., “I feel overwhelmed by my work,”) cynicism (e.g., “I feel lack of motivation in my work and often think of giving up,”) and sense of inadequacy (e.g., “I often have feelings of inadequacy in my work.”) The scale anchors were (1 = completely disagree; 6 = completely agree). A mean score was formed to concern principals’ overall burnout (Cronbach’s α = 0.90) (see also Schaufeli et al., 2002). *Job engagement* was measured with a short version of the Utrecht Work Engagement Scale, UWES-S (Schaufeli et al., 2002; see also Schaufeli et al., 2006; Toth-Kiraly et al., 2021a,b). The scale consists of 9 items measuring energy (e.g., “When I work, I am bursting with energy,”) dedication (e.g., “I am enthusiastic about my work”), and absorption (e.g., “Time flies when I’m working”). The responses were rated on a 7-point scale (1 = never; 7 = daily). A mean score was formed to measure principals’ overall engagement at work (Cronbach’s α = 0.94) (see also Schaufeli et al., 2006).

### Analysis Strategy

To be able to identify the homogeneous latent groups of school principals with different levels of stress sources (e.g., stress about students’, teachers’, parents’, and principals’ own ability to cope with the situation) during the COVID-19 pandemic, the results were analyzed by means of latent profile analysis (LPA; Muthén and Muthén, 1998–2021), which is a type of finite mixture analysis that assesses heterogeneity through the identification of homogeneous subgroups (i.e., latent profiles) of participants with similar indicator means (e.g., principals’ stress sources) within the latent profiles. The advantage of LPA compared with traditional cluster analysis is that it is model-based and provides fit indices for different latent profile solutions, which can then be compared in order to determine the final solution which fits the data the best (see also Mäkikangas et al., 2018). The latent profile analyses were carried out in two phases. First, to be able to identify naturally occurring latent profiles of principals’ stress concerning the school community’s ability to cope in the data, LPAs for different latent groups were carried out first, and the fit indices and class frequencies were compared. The variances were estimated equal between the classes by default. The estimation was performed step by step, starting from one-class solution to estimate the parameters for 2,3,…, *k*-class solutions. The solution that best fitted the data in accordance with the indicators and that was also deemed reasonable in terms of interpretation was chosen as the final latent profile model. Second, in order to identify the possible antecedents of principals’ stress profiles, job demands (e.g., workload, remote work stress, difficulty to detach from work, COVID-19 crisis, COVID-19 infections at school, impact of COVID-19 on future teaching), resources (e.g., buoyancy, crisis leadership, social appreciation, schools’ adaptation to remote learning), and job burnout and engagement were added into the final model as covariates using multinomial logistic regression via the R3STEP command (Asparouhov and Muthén, 2014). The R3STEP provides information whether the antecedent variables are related to a higher probability of the participants belonging to each profile rather than the others (see Table 1 for means, standard deviations, and correlations).

**TABLE 1:** Correlation coefficients, means, and standard deviations.

	1	2	3	4	5	6	7	8	9	10	11	12	13	14	15	16
1. Students’ coping																
2. Teachers’ coping	0.61***															
3. Parents’ coping	0.52***	0.62***														
4. Principals’ coping	0.30***	0.55***	0.32***													
5. Job burnout	0.18***	0.35***	0.17***	0.58***												
6. Job engagement	–0.08	−0.18***	–0.03	−0.38***	−0.61***											
7. Buoyancy	−0.16***	−0.28***	−0.12**	−0.51***	−0.58***	0.46***										
8. Crisis leadership	−0.12**	−0.12**	–0.02	−0.09*	−0.14***	0.21***	0.26***									
9. Social appreciation	0.04	−0.16**	0.01	−0.19***	−0.39***	0.42***	0.29***	0.27***								
10. Adaptation to remote learning	−0.10*	−0.12**	–0.05	−0.15***	−0.23***	0.24***	0.22***	0.20***	−0.32***							
11. Workload	0.20***	0.40***	0.21***	0.57***	0.48***	−0.27***	−0.36***	−0.09*	0.21***	−0.09*						
12. Remote work stress	0.20***	0.26***	0.28***	0.35***	0.24***	−0.12**	−0.18***	−0.10*	0.13**	−0.15***	0.37***					
13. Difficulty to detach	0.15***	0.27***	0.18***	0.51***	0.53***	−0.25***	−0.47***	−0.11*	0.15***	–0.07	0.41***	0.31***				
14. COVID-19	0.15***	0.16***	0.20***	0.17***	0.14**	–0.05	−0.10*	–0.02	0.06	–0.08	0.13**	0.27***	0.15***			
15. COVID-19 infections	0.11*	0.18***	0.20***	0.17***	0.13**	–0.00	–0.08	0.01	0.10*	–0.06	0.11*	0.20***	0.14**	0.76***		
16. Impact of COVID-19 on future teaching	0.16***	0.19***	0.19***	0.20***	0.14**	–0.08	−0.09*	0.02	0.00	–0.06	0.09*	0.21***	0.15***	0.30***	0.31***	
*M*	5.49	6.97	5.20	5.65	2.72	5.16	4.89	3.88	1.73	4.03	7.42	4.72	3.97	6.01	6.43	6.37
*SD*	2.41	2.18	2.58	2.74	0.78	1.01	1.17	0.57	0.48	0.50	2.28	2.99	2.75	2.58	2.21	2.27

***p < 0.001; **p < 0.01; *p < 0.05; Response scales, 1–4 social appreciation; 1–5 COVID-19 crisis leadership efficacy, adaptation to remote learning; 1–7 job burnout and engagement, buoyancy; 0–10 remote work stress, COVID-19 infections at school, impact on teaching; 1–10 students’, teachers’, parents’, principals’ coping, difficulty to detach, workload, COVID-19 crisis.

All the analyses for the LPAs were performed with the Mplus statistical package (version 8; Muthén and Muthén, 1998–2021). Missing data was deleted listwise, which was the default for this type of analysis (Muthén and Muthén, 1998–2021). The model parameters were estimated by means of maximum likelihood robust (MLR) estimator, which produces standard errors and a chi-square test statistic for missing data with non-normal outcomes by means of a sandwich estimator and the Yuan-Bentler T2 test statistic (Muthén and Muthén, 1998–2021). Five criteria were used to decide the final number of classes: (a) the Bayesian information criterion (BIC), and (b) the Akaike information criterion (AIC), according to which the model with the smallest value is considered the best model; (c) the Vuong-Lo-Mendell-Rubin (VLMR) test of fit, which compares solutions with different numbers of profiles (a low *p*-value indicates that the *k* model has to be rejected in favor of a model with at least *k* + 1 profiles); (d) entropy values, which determine classification quality (values close to 1 indicate clear classification) (Celeux and Soromenho, 1996); and (e) the clarity and interpretation of the profiles.

## Results

**FIGURE 2:**
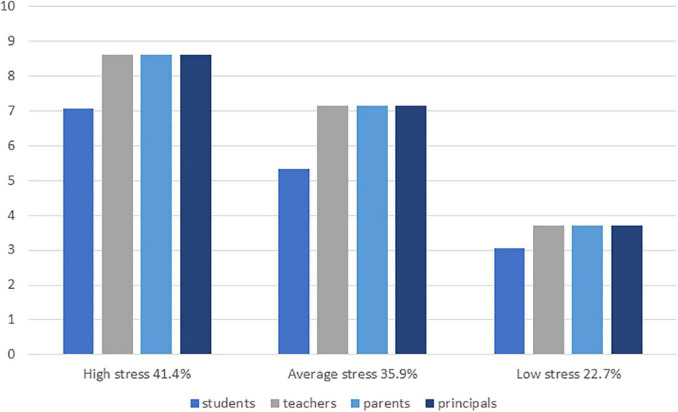
Latent profiles of school principals’ stress concerning students’, teachers’, parents’, and principals’ ability to cope during the COVID-19 pandemic.

The analyses were begun by performing LPAs with different numbers of latent profiles. Table 2 shows the different fit indices for the compared latent profile solutions. Comparison of the fit indices and profile frequencies showed that the fit indices of three and four profile solutions were quite similar and both solutions would fit the data well. However, a closer examination of the profiles indicated that in the only difference between three- and four profile solutions was that in the four profile solution the largest group split in two smaller groups which were highly similar in terms of principals’ stress concerning the school community’s ability to cope. Thus, the three-profile solution was considered the most distinctive and meaningful solution. The final three-profile solution is presented in Figure 2.

**TABLE 2:** Fit indices for the compared latent profiles.

Number of profiles	BIC	aBIC	AIC	Entropy	VLMR	Difference in the number of parameters	*p*-value	Latent class proportion%
1	9983.39	9957.10	9949.14	−	−	−		
2	9383.76	9342.50	9328.09	0.88	−4966.57	5	0.00	70/30
3	9215.92	9158.79	9138.84	0.82	−4651.05	5	0.00	41/36/23/
4	9140.56	9067.55	9042.07	0.83	−4551.42	5	0.00	35/23/22/20
5	9131.26	9042.38	9011.35	0.80	−4498.04	5	0.08	26/22/21/20/11

*BIC, Bayes information criteria; aBIC, Adjusted Bayes information criteria; AIC, Akaike information criteria; VLMR, Vuong–Lo–Mendell–Rubin.*

The first latent profile (41.4% of the principals) was characterized by a high level of stress related to parents, teachers, and principals’ own ability to cope during COVID-19, and altered level of stress related to students’ ability to cope (Figure 2). The second latent profile (35.9% of the principals) was characterized by an altered level of stress related to parents, teachers, and principals’ own ability to cope, and average level of stress related to students’ ability to cope. The third latent profile (22.7%) was characterized by a low level of all the variables. The latent profiles were labeled as *high, altered, and low stress* profiles.

Next, to investigate the role of covariates in predicting the latent profiles, principals’ job resources (e.g., buoyancy, crisis leadership, social appreciation, schools’ adaptation to remote learning), job demands (e.g., workload, remote work stress, difficulty to detach from work, COVID-19 crisis, COVID-19 infections at school, impact of COVID-19 on future teaching), and job burnout and engagement were added in the final model. The results for the covariates (Table 3) showed that school principals who reported having high job resources (e.g., buoyancy, social appreciation) more often belonged to low rather than high or average stress profiles. In addition, principals who thought the transition to remote learning was successful at their school more often belonged to the average rather than to the high stress profile. Principals who experienced they were able to perform their leadership tasks well during the COVID-19 crisis more often belonged to the average rather than to the two other profiles. Concerning demands, principals who experienced high job and personal demands (e.g., workload, stress related to remote work, difficulty to detach from work), more often belonged to the high or average stress profiles rather than the low stress profile, or to high rather than to the average stress profile. Further, principals who experienced high stress related to COVID-19 pandemic, infections spreading at school, and concerning the impact of COVID-19 on future teaching more often belonged to the high rather than to the low or average stress profiles. Moreover, principals who experienced high job burnout more often belonged to the high or average stress profile rather than to the low stress profile, whereas principals who experienced high work engagement more often belonged to the low stress rather than to the high or average stress profiles.

**TABLE 3:** Antecedents of school principals stress source profiles (logistic regression coefficients).

	High vs. Low	High vs. Average	Average vs. Low
**Job well-being**			
Burnout	1.34***	0.25	1.09***
Engagement	−0.48**	0.01	−0.49***
**Resources**			
Buoyancy	−0.74***	0.10	−0.68***
Social appreciation	−0.70*	0.24	−0.73*
COVID-19 leadership	–0.00	−0.60**	0.60*
Adaptation to remote learning	–0.27	−0.57*	0.30
**Demands**			
Workload	0.49***	0.17**	0.31***
COVID-19 crisis leadership	1.56***	1.28***	1.21***
Remote work stress	0.27***	0.19***	0.08
Difficulty to detach from school	0.29***	0.07	0.23***
COVID-19	0.19***	0.16**	0.03
COVID-19 infections at school	0.26***	0.16**	–0.10
Impact of COVID-19 on future teaching	0.25***	0.14*	0.11

***p < 0.001; **p < 0.01; *p < 0.05.

## Discussion

The present study examined latent profiles of stress among Finnish school principals during an unprecedented time of global COVID-19 pandemic, when in many countries lockdowns, school closures, and social distancing took place, resulting as increased levels of stress, anxiety, and loneliness among students (Bu et al., 2020; Zheng et al., 2021), which had detrimental effects on some students’ well-being (Loades et al., 2020; Salmela-Aro et al., 2021). Teachers and school principals were forced to engage in a dramatically different way, and shift from in-person to remote work (Weiner et al., 2021). Some (mainly primary) schools faced another shift at the end of the spring 2020, when after the initial closures schools went back to in-person teaching, which continued also during the next school term starting in autumn 2020. School principals were facing unexpected challenges and leading rapid shifts between in-person teaching and virtual teaching and learning, while concerns about students’, teachers’, parents’, and principals’ own ability to cope with the situation were simultaneously present. This study examined latent profiles of principals’ stress concerning the school community’s ability to cope during the pandemic in association with multiple job demands and resources during COVID-19.

### Latent Profiles of Principals’ Stress

Three distinct latent profiles concerning school principals’ stress related to students’, teachers’, parents’, and their own ability to cope during COVID-19 were identified, namely high, average, and low stress profiles. The two largest profiles were the high (41.4%) and average (35.9%) stress profiles, which were characterized by a high/average level of stress related to parents, teachers, and principals’ own ability to cope, and altered/average level of stress related to students’ ability to cope. The third “low stress” profile (22.7%) was characterized by an overall low level of stress. These alarming results indicated that during COVID-19, 77% of the principals were experiencing high or altered levels of stress concerning the school community’s ability to cope with the pandemic. The pandemic has altered the nature of school principals’ work, and principals are extending their roles to create safe school settings for now and future education, provide tools and support for virtual teaching (managing physical distance, establishing effective communication strategies, motivating staff, establishing trust), and answer to the concerns and worries of the school community (Pollock, 2020). Teachers’ and principals’ work intensified especially at the beginning stages of the pandemic when the present study was also conducted. Due to the school- and workplace closures, many parents had to supervise their children’s schooling at home while simultaneously managing their own work remotely, and many students reported increasing anxiety and loneliness (Bu et al., 2020; Zheng et al., 2021). All these sudden turmoils in school community manifested as altered levels of stress among school principals.

According to the appraisal approach, stressors are not direct precipitating causes of stress reactions, it is rather the person’s appraisals of the stressors which determine their responses (Storch et al., 2007; Giancola et al., 2009; Webster et al., 2011). Acute stress typically increases engagement and coping with the stressful event or situation which is the cause (Von Dawans et al., 2012). However, the strain is likely to cease once the exposure to the stressors are removed, or, if the situation persists, people will continue to experience strain and other negative reactions such as sustained fatigue (Webster et al., 2011). In the current situation it is difficult to quantify the possible consequences of the pandemic on student achievement and well-being (Kuhfeld et al., 2020), as well as the possible long-term effects on education. It is likely that principals’ stress about school community’s ability to cope continues for some time, however, it is possible to promote school communities’ resilience already during the pandemic, for example, by addressing the possible inequalities and decreases in well-being early (Sahlberg, 2021).

Interestingly, within each profile, principals were slightly less concerned about students’ ability to cope compared to adults, which may reflect the burden teachers, parents, and principals were facing while managing online teaching of the students. However, it is possible that when the pandemic prolonged, and school societies became more aware of the negative effects of the pandemic on student well-being (Bu et al., 2020; Zheng et al., 2021), related concerns increased. On the other hand, as the crisis continued, students, teachers, parents, and principals acquired new skills and found better ways to adapt, which may also have showed as decreases in principals’ stress. More studies would be needed to examine the possible changes in principals’ stress profiles during different phases of the ongoing pandemic further.

### Demands and Resources Associated With Principals’ Stress

The results indicated that high buoyancy as a personal resource was associated with lower levels of school principals’ stress concerning the school community’s ability to cope. Buoyancy refers to one’s “everyday” resilience, and capacity to overcome challenges at work, and is often associated with high well-being among employees (Martin and Marsh, 2008a,b; Parker and Martin, 2009). Capability to lead school effectively during the pandemic was associated with average stress, probably because times of crisis are always stressful. Too high or low levels of stress may hinder effective crisis leadership (see also DeMatthews et al., 2021). Similarly, principals who felt their school’s transition to remote learning went well more often belonged to the average rather than to the high stress profile. Further, principals who experienced high social appreciation more often belonged to the low stress profile compared to the other two profiles. Social support from the school community is often associated with lower levels of occupational stress, and principals who experience social support feel more connected to school community (Beausaert et al., 2016). In addition, occupational well-being in terms of job burnout and engagement was associated with high/altered stress or low stress, respectively.

Job/personal (workload, remote work stress, strain caused by the COVID-19 crisis) and social demands (COVID-19 infections at school, impact of COVID-19 on future teaching) were associated with high or altered levels of stress concerning the school community’s ability to cope. In the crisis situation, principals were rapidly taking new tasks, such as creating effective and safe learning environments for students and teachers, which likely increased their workload. Simultaneously other aspects of COVID-19 crisis (concerns and fears about the virus spreading, reduced social contacts, multitasking with remote work and family duties) were present, manifesting as altered stress. Further, these results indicated that it was not only COVID-19 related social demands which altered principals’ stress, but also other job/personal demands, such as workload and remote work stress, which increased principals’ stress concerning their school community’s ability to cope. The pandemic has altered the nature of school principals’ work, and principals are extending their roles to create safe school settings for now and future education, provide tools and support for virtual teaching (managing physical distance, establishing effective communication strategies, motivating staff, establishing trust), and answer to the concerns and worries of the school community (Pollock, 2020). Teachers’ and principals’ work intensified especially at the beginning stages of the pandemic when the present study was also conducted.

## Limitations

This study has some limitations which should be taken into account when generalizing the findings. First, the study design was cross-sectional, which made it not possible to examine the development of principals’ stress across the pandemic. Due to the multiple changes in the school environment, it is possible that increases/decreases occurred in principals’ stress. Such development should be examined in future studies using longitudinal designs. Second, this study concerned Finnish school principals whose job is characterized by high independency and flexible accountability (Aho et al., 2006). It is possible that the results would have turned different in some other cultural contexts and occupational groups, and more studies would be needed to examine such differences further. Third, some variables which were not examined in this study might have affected the results (e.g., students’ disruptive behaviors, job-related self-efficacy) (Upadyaya et al., 2016; Bottiani et al., 2019). Moreover, besides job burnout and engagement, principals’ occupational well-being covers multiple other constructs (Kowalski and Loretto, 2017), such as job satisfaction, which were not examined in the present study. In the future studies, it would be important to examine multiple types of demands, resources, and indicators of well-being in association with principals’ stress profiles.

## Conclusions

Compared to teachers, principals are more likely to experience occupational stress (Darmody and Smyth, 2011). The present study showed some concerning results by indicating that during COVID-19, most school principals (77%) experienced high or altered levels of stress related to the school community’s ability to cope with the COVID-19 crisis. Principals’ job/personal/social demands and resources, burnout and engagement were associated with their experiences of stress. Especially buoyancy and social support (appreciation) from school community were beneficial in protecting principals against high stress. These results are important, as principals’ stress, well-being, and leadership style can be associated with workplace buoyancy among teachers (Collie et al., 2016), and thus, have manifold influence in the school community. Social support is an important job resource which may further manifest as high well-being and connectedness, and buffer against the negative impact of job demands (Bakker et al., 2007; Beausaert et al., 2016). Social support also helps in taking care of work burden in daily tasks (Beausaert et al., 2016). Poor social support, in turn, is often associated with higher levels of occupational stress among principals (Darmody and Smyth, 2011). Principals’ experiences of stress concerning the school society’s coping with the pandemic are unique in a sense that principals are the ones who are responsible of leading the school, responding to the changes in the crisis situation, and providing the necessary measures for the school community’s adjustment. As the pandemic is still ongoing, and principals are continuously required to respond to the new regulations, and build safe environments to school communities, it would be important to simultaneously promote principals’ personal and social resources, and reduce their job demands. For example, in order to reduce principals’ stress and workload, it would be possible to share some of principals’ job responsibilities with other colleagues or an administrative team (Beausaert et al., 2016). Principals’ job demands have been steadily increasing, and the unpredicted changes the pandemic caused created new demands and stress, as the effort required to meet demands cannot always be effectively directed, or the quantity of demands is high (Beausaert et al., 2021). Promoting collegiality and collaboration in principals’ work would help in creating social capital, which support principals’ well-being (Beausaert et al., 2021). Coaching and mentoring may also provide social support, help principals to feel less isolated, and mitigate the overload principals may experience during crisis situation (Bauer and Brazer, 2013).

## Data Availability Statement

The raw data supporting the conclusions of this article will be made available by the authors, without undue reservation.

## Ethics Statement

The studies involving human participants were reviewed and approved by University of Helsinki Ethics Committee. Written informed consent for participation was not required for this study in accordance with the national legislation and the institutional requirements.

## Author Contributions

KU contributed to the writing of the manuscript and performed the statistical analyses. KS-A contributed to the design of the study, data collection, and writing of the manuscript. HT contributed in editing and writing the manuscript. All authors contributed to the article and approved the submitted version.

## Conflict of Interest

The authors declare that the research was conducted in the absence of any commercial or financial relationships that could be construed as a potential conflict of interest.

## Publisher’s Note

All claims expressed in this article are solely those of the authors and do not necessarily represent those of their affiliated organizations, or those of the publisher, the editors and the reviewers. Any product that may be evaluated in this article, or claim that may be made by its manufacturer, is not guaranteed or endorsed by the publisher.
